# Regulatory Actions of Estrogen Receptor Signaling in the Cardiovascular System

**DOI:** 10.3389/fendo.2019.00909

**Published:** 2020-01-10

**Authors:** Kazutaka Ueda, Yusuke Adachi, Pangyen Liu, Nobuaki Fukuma, Eiki Takimoto

**Affiliations:** ^1^Department of Cardiovascular Medicine, Graduate School of Medicine, The University of Tokyo, Tokyo, Japan; ^2^Division of Cardiology, Department of Medicine, Johns Hopkins University School of Medicine, Baltimore, MD, United States

**Keywords:** estrogen, cardiovascular diseases, receptor signaling, non-nuclear pathway, animal study

## Abstract

Premenopausal females have a lower incidence of death from cardiovascular disease (CVD) than male counterparts, supporting the notion that estrogen is protective against the development and progression of CVD. Although large-scale randomized trials of postmenopausal hormone replacement therapy failed to show cardiovascular benefits, recent ELITE study demonstrated anti-atherosclerotic benefits of exogenous estrogen depending on the initiation timing of the therapy. These results have urged us to better understand the mechanisms for actions of estrogens on CVD. Here, we review experimental and human studies, highlighting the emerging role of estrogen's non-nuclear actions linking to NO-cGMP signaling pathways.

## Introduction

In developed countries, cardiovascular diseases (CVD) are the leading cause of death in males and females. As females are less likely to develop CVD before menopause, the endogenous female hormone estrogen appears to provide protection against CVD (1). Consistently, numerous experimental studies of CVD models affirmed the beneficial effects of estrogen treatment, including the inhibition of the development of atherosclerosis and endothelial dysfunction, and the reduction in myocardial ischemic injuries (2–4). While earlier large-scale randomized clinical studies of postmenopausal hormone replacement therapy showed rather adverse events without significant cardiovascular benefits (4, 5), a recent clinical study revealed that the timing for therapy initiation might be a critical determination factor (6).

Estrogen binds to multiple receptors, including classical nuclear estrogen receptors (ERs), ERα, and ERβ, and a new class of membrane G protein-coupled receptor, GPR30, also referred to as GPER (7). The ERs signal not only via “classical” regulation of gene transcription in the nucleus, but also via regulation of “non-nuclear” signaling pathways on ligand binding to ERs (1, 8). Accumulating evidence has shown a critical role for the non-nuclear ER signaling in maintaining the homeostasis of the cardiovascular system (9).

## The Effects of Estrogen Hormone Therapy on the Cardiovascular System

Multiple cohort studies consistently revealed the lower risk of CVD with hormone therapy (HT), indicating that estrogen loss enhances the risk of CVD in postmenopausal females (2–4). On the other hand, randomized studies including the Women's Health Initiative (WHI) showed no cardiovascular benefits, but rather found an increased risk of stroke and deep vein thrombosis (4, 5). These discordant findings could reflect variations in the time between menopause and HT initiation: earlier cohort studies involved younger women who received HT early after menopause, and patients in randomized studies receive HT 10 years after menopause, where vascular endothelium might have lost its responsiveness to estrogen.

Indeed, several recent studies provided supporting evidence for this “timing hypothesis.” Estrogen alone reduced total mortality in early (<10 years) postmenopausal females (10). After 10 years of randomized treatment, women who received HT early after menopause showed a significantly reduced risk of mortality, heart failure, and myocardial infarction, without increases in the risk of cancer, venous thromboembolism, or stroke (11). The Early vs. Late Intervention Trial with Estradiol (ELITE) study enrolled postmenopausal females with and without histories of prior hysterectomy surgery (6). Approximately half of the participants were considered early menopause (<6 years; mean age, 55 years), and the other half were later menopause (at least 10 years; mean age, 65 years). Participants were randomized to receive either oral 17 β-estradiol or a placebo, and the carotid intima-medial thickness (CIMT) by ultrasound, an atherosclerosis marker predicting cardiovascular events, was assessed as the primary clinical outcome. Over 6 years, the early menopause participants who were randomized to 17 β-estradiol showed a slower progression of CIMT than those randomized to placebo (6). These results strongly indicate that HT exerts cardioprotective effects when initiated at an ideal time point after menopause.

These recent clinical findings encourage researchers to further investigate molecular and physiological function of ER-mediated signaling in the cardiovascular system (12, 13), which provides meaningful steps forward to the development of a next generation of HT for women that capture the beneficial cardiovascular effects of the hormone while minimizing its potential for harm.

### Estrogen Receptors and Cardiovascular Cells

**Figure 1:**
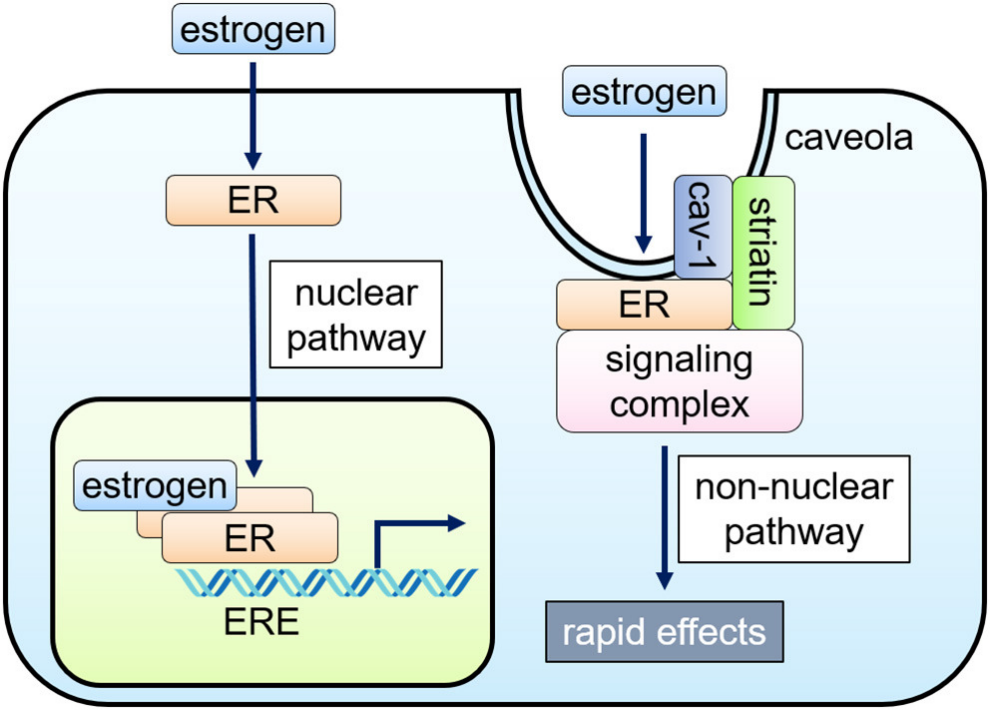
Nuclear and non-nuclear ER signaling pathways. Classically, the hormone-bound estrogen receptor (ER) dimerizes and binds to specific DNA sequences called estrogen response elements (ERE) and activates gene expression (nuclear pathway). Alternatively, ERs localized to caveolae, cell membrane microdomains, can signal without nuclear translocation through inducing a subpopulation of cell membrane–associated ERs to form a signaling complex that results in rapid activation of specific kinases and eNOS in endothelial cells (non-nuclear pathway).

ERα and ERβ exhibit high homology and, as with all steroid hormone receptors, function as transcription factors that alter gene expression when activated (3). Functional ERs are expressed in vascular endothelial cells (EC), vascular smooth muscle cells (VSMC), and cardiomyocytes in humans and animals (3) (Figure 1). Some cardiovascular effects might be mediated by a transmembrane G-protein-coupled receptor GPR30 which is extensively reviewed elsewhere (7, 14), and thus this mini review will focus on ERα and ERβ.

### Nuclear ER Signaling

In the nucleus, ligand-bound ERs interact with estrogen response elements (ERE) and function as transcription factors, regulating gene expression (1). Nuclear ER-estrogen complexes also modulate the function of other classes of transcription factors through protein-protein interactions; thus, controlling gene expression without direct binding to DNA (1). Cell type-specific recruitment of co-activators and displacement of co-repressors to DNA binding sites determine cellular response to estrogen (3, 15).

### Non-nuclear ER Signaling

Cellular responses to estrogen occur within minutes, which are mediated via enzymatic pathways through the activation of membrane-associated ER, referred to as “rapid” or “non-nuclear” ER signaling (9, 16). Non-nuclear ER signaling has been documented in a variety of cell types *in vitro*, including oocytes, osteoblasts, osteoclasts, breast cancer cells, adipocytes, VSMC, EC, and cardiac myocytes (17–19). The rapid actions originate at the ERs located at caveolae, small invaginations of the cell membrane, activating kinases, or phosphatases to impact cell physiology (16, 20–22).

The non-nuclear ER signaling in the cardiovascular system has been most studied in EC where rapid eNOS activation by estrogen within 15–30 min was initially identified (23, 24). ERα, co-localized with caveolin-1, binds to a scaffold protein striatin, also associated with caveolin-1 (20, 25, 26). Endogenous ERβ was also detected in the EC membrane, particularly at the caveolae (27), but its associated proteins remain undetermined.

ERs-localized to caveolae activate PI3K, Akt kinase, and ERK1/2, enhancing phosphorylation of Ser-1177 of eNOS (22, 28–30). The activation of PI3K involves a complex process. ERα directly binds to the p85α regulatory subunit of PI3K in a ligand-dependent fashion (30), while PI3K activation requires c-Src kinase, whose SH2 domain interacts with phosphorylated Tyr-537 of ERα (31, 32). Gαi is also involved in this ERα complex at the caveolae, and the physical association of ERα with Gαi is required for eNOS activation (33, 34). On the other hand, striatin serves as a scaffold protein of the ERα complex at the caveolae. Since interruption of ERα-striatin binding, either with a peptide representing ERα amino acids 176-253 or the ERα triple point mutation (lysine 231, arginine 233, and arginine 234 to alanine: KRR), leads to defective non-nuclear signaling without affecting nuclear signaling, striatin also plays a key role to the non-nuclear signaling of ERα (25, 35, 36).

### Estrogen-Independent ER Signaling

Interestingly, unliganded ERs or growth factor signaling-mediated ERs play a role in cellular physiology in the absence of estrogen. The presence of unliganded ERα alone decreases EC migration and EC proliferation, and increases SMC proliferation (37). Unliganded ERα also regulates gene expression of ECs. These effects either on physiology or gene expression are reversed by ERα activation in the presence of E2 (37). ERs can be activated in the absence of estrogen by growth factor receptor signaling such as epidermal growth factor and insulin-like growth factor receptors (38), and such estrogen-independent ER activation has been reportedly induced by different intercellular pathways in vascular as well as non-vascular cells (3, 16). Thus, cardioprotective effects of estrogen might be in part attributable to preventing the adverse cardiovascular effects of unliganded ERα by its binding to the receptor and other growth factor signal-mediated ERα activation.

## Actions of Estrogen in Cardiovascular Cells

Functional ERs are expressed in multiple cell types which compose the heart. Estrogen regulates diverse cellular functions via nuclear and non-nuclear signaling pathways or ligand-independent signaling pathways; estrogen exerts inhibition of cellular hypertrophy and apoptosis in cardiomyocytes, proliferation of cardiac fibroblasts, proliferation and eNOS signaling activation in vascular endothelium, and anti-proliferative effect on vascular smooth muscle cells. In this section, we review cell-type specific *in vitro* studies, including cardiomyocytes, cardiac fibroblasts, vascular endothelial, and smooth muscle cells.

### Cardiomyocytes

**Figure 2:**
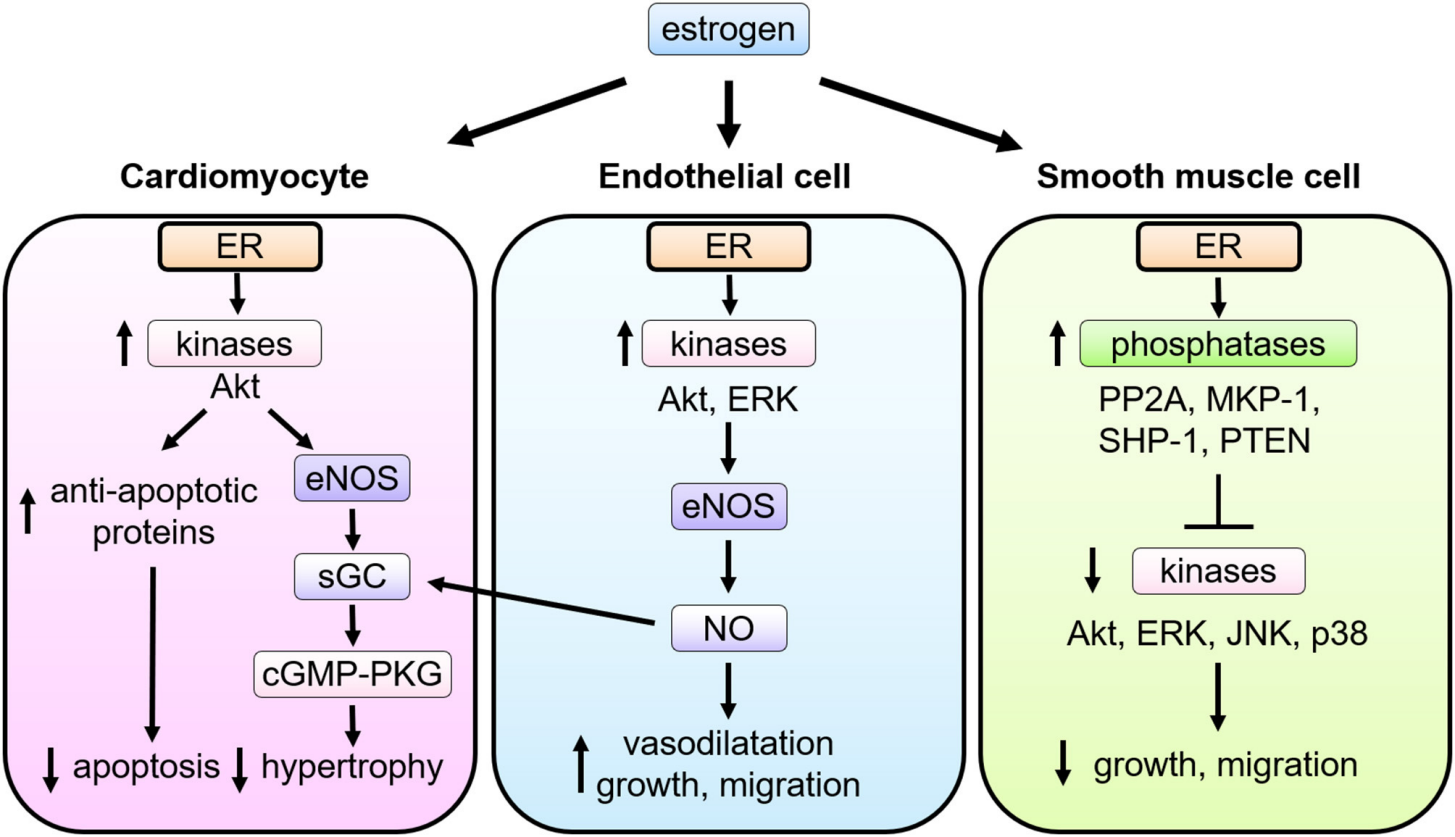
Multiple actions of estrogen-estrogen receptor signaling in cardiovascular cells. In cardiomyocytes, ER signaling leads to the phospho-activation of Akt and eNOS, the latter stimulating soluble guanylate cyclase (sGC) to activate cyclic guanosine monophosphate (cGMP)—PKG (cGMP-dependent protein kinase) signaling pathways and thereby attenuate pathological cardiac hypertrophy. In endothelial cells (EC), upon estrogen binding, ER signaling complex activate the tyrosine kinase Src, the serine/threonine kinase PI3K, and the kinase Akt. Akt then directly phosphorylates eNOS on serine 1177, leading to its enzymatic activation and production of NO, which promotes EC proliferation and migration as well as vasodilatation. NO produced in EC also functions as a stimulator of sGC in cardiomyocytes. In smooth muscle cells (VSMC), upon stimulation of estrogen, ER interacts with and activates several phosphatases including protein phosphatase 2A (PP2A), mitogen-activated protein kinase phosphatase 1 (MKP-1), Src homology region 2 domain-containing phosphatase-1 (SHP-1), and phosphatase and tensin homolog deleted from chromosome 10 (PTEN), and modulates activation of kinases induced by growth stimulation, leading to inhibition of VSMC proliferation and migration.

Cardiomyocytes express functional ERs, and estrogen regulates expression of cardiac genes, such as connexin 43, β-myosin heavy chain and ion channels (39, 40). Estrogen also regulates calcineurin abundance, cGMP-PKG activation, Akt activation, and miRNAs in cardiomyocytes to inhibit cellular hypertrophy and confer protection against apoptosis, where both nuclear and non-nuclear pathways might be involved (Figure 2). Sasaki et al. demonstrated rapid activation of cGMP-PKG in response to estrogen in cardiac myocytes, providing the link between estrogen and cGMP-PKG signaling, both of which are known to play protective roles against diverse cardiac pathologies, including cardiac hypertrophy, failure, and ischemic injuries (41). While cGMP-PKG inhibits activation of calcineurin, a central molecule for pathological hypertrophy, estrogen also enhances the degradation of calcineurin. Estrogen activates Akt and inhibits apoptosis via rapid signaling, while it down-regulates miR22 to activate Sp1 for anti-oxidant induction (42–44). Furthermore, Lin et al. reported that estrogen and ERβ-specific agonist diarylpropionitrile increased S-nitrosylation of heat shock proteins and decreased infarct area after ischemia-reperfusion in isolated mice hearts, while these effects were abolished in ERβ knockout mice or with a NOS inhibitor (45).

### Cardiac Fibroblasts

Cardiac fibroblasts are the primary source of myocardial extracellular matrix proteins, matrix metalloproteinases (MMPs), growth factors, and cytokines, all of which contribute to cardiac remodeling on physiological and pathological levels (46). Lee and Eghbali-Webb reported that both ERα and β are expressed in fibroblasts isolated from female rats, where ERβ is predominant with both cytosolic and nuclear localization (47). They also reported that 17 β-estradiol enhances cardiac fibroblast proliferation through mitogen-activated protein kinase-dependent mechanisms (47).

### Vascular Endothelial Cells

NO derived from eNOS activation exerts potent cardiovascular effects, including vascular relaxation, growth and migration of ECs and VSMCs, and the antagonism of platelet activation, thrombus formation, and leukocyte-EC adhesion (27, 29, 48–50). Estradiol conjugated to bovine serum albumin (E2-BSA), which does not pass through the cell membrane, was originally used to evaluate the role of cell membrane ER (51), and shown to promote phosphorylation of eNOS in cultured EC. Consistently, an estrogen-dendrimer conjugate (EDC) that activates membrane-associated ER, but is excluded from the nucleus (52), was shown to stimulate cultured EC proliferation and migration via ERα, heterotrimeric G protein Gi, and the activation of eNOS (53) (Figure 2).

Recent transcriptome analysis by Lu et al. using EC expressing KRR mutant ER revealed that estrogen regulates 60 genes in ECs expressing wild-type ERα, many of which were associated with cell migration and proliferation identified by pathway analysis. Meanwhile, only 10 genes were regulated by E2 in ECs expressing KRR ERα and thus lacking non-nuclear signaling pathway. These data provide the evidence that non-nuclear signaling also plays a pivotal role for the transcriptional responses of EC to estrogen (35).

### Vascular Smooth Muscle Cells

Estrogen exerts anti-proliferative effects on VSMC (54–56). Cell proliferation is considerably regulated by kinase-mediated signal transduction. Treatment with estrogen inhibits phosphorylation of growth-related kinases, such as ERK1/2, JNK, p38, and Akt, which are phosphorylated and activated by growth factor stimulation (54, 55). Estrogen controls the expression and activity of several phosphatases in VSMC (57, 58), including MKP-1, SHP-1, PTEN, and PP2A, balancing the kinases (56, 59, 60). In cultured VSMCs, ERα and PP2A form a complex, and estrogen treatment increases PP2A activity resulting in the inhibition of growth-promoting signal activation (61). The role of non-nuclear ER signaling in VSMC proliferation was evaluated with using a transgenic mouse model (Disrupting Peptide Mouse; DPM), in which non-nuclear ER-mediated signaling was abolished by overexpression of the peptide representing amino acids 176–253 of ERα, preventing ER from forming a signaling complex with striatin (62). Estrogen inhibition on VSMC proliferation was lost in VSMC derived from the DPM (61), supporting the theory that the non-nuclear ER signaling pathway is required for the estrogen-mediated anti-proliferative effects in VSMC (Figure 2).

## Estrogen Actions in Animal Models of Cardiovascular Diseases

To date, many studies using experimental models of CVD have been reported to elucidate the role of estrogen in the cardiovascular system. Most studies utilized pharmacological interventions or global gene deletion, whereas studies employing tissue-specific mutant models would provide more insights into our understanding the role of estrogen in CVD.

### Ischemic Heart Diseases

ERα and ERβ are both reportedly involved in the cardioprotective effects of estrogen. Global ERβ KO mice revealed increased mortality and exacerbated heart failure after myocardial infarction (63). Consistently, cardiomyocyte-specific ERβ overexpression improved cardiac function and survival after myocardial infarction induced by left anterior descending coronary artery ligation. On the other hand, cardiac fibrosis post-myocardial infarction was attenuated with increased angiogenesis in female mice overexpressing ERα (64, 65).

In an ischemia-reperfusion model of ovariectomized global ERα KO mice, coronary endothelial dysfunction was not reversed by estrogen, while it was normalized in wild types (66). Other studies further revealed ERα KO demonstrated exacerbated ischemia reperfusion phenotype, including markedly impaired cardiac contractility, increased cardiomyocyte death, and mitochondrial damage (67, 68). On the other hand, ERβ KO female hearts exhibited poor functional recovery compared to wild-type or ERα KO mice in an *ex vivo* model of global ischemia-reperfusion (69). Mechanistically, estrogen mitigates reperfusion injuries after ischemia primarily by activating PI3K-Akt, increasing expression of the anti-apoptotic protein BCL-2 and decreasing the expression of the proapoptotic caspase proteins (43). Importantly, neither ischemic recovery nor PI3K-Akt activation was observed in hearts isolated from female ERβ KO mice (43, 70, 71). Together, ERβ appears to play substantial cardioprotective roles against ischemia reperfusion injury, while the role of ERα seems to differ depending on methodological conditions.

Estrogen might contribute to regenerative process in the heart. Cardiac stem cells treated with estrogen produce protective factors and improve cardiac function and cardiomyocyte survival when injected into hearts after ischemia-reperfusion injury (72). Both ERα and β contribute to estrogen-mediated endothelial progenitor cell activation and mobilization in tissues, helping preserve cardiac function after myocardial infarction (73). ERα selective agonist propylpyrazoletriol reduced apoptosis and increased survival of cardiac cells expressing c-Kit, a potential marker for adult cardiac stem cells, in infarcted hearts; whereas the ERβ selective agonist diarylpropionitrile had no effect (74).

### Cardiac Hypertrophy and Failure

The heart develops pathological hypertrophy in response to various pathological stressors, such as genetic, mechanical, or excessive neurohormonal stress. If the stress is sustained, hypertrophy transits to failure. Sex difference has been known to be a modifier of human cardiomyopathy where estrogen also plays a role (75). Genetically-modified mouse models of hypertrophic cardiomyopathy presented sex differences of cardiac phenotype. Mice with a missense mutation (R403Q) in the α-myosin heavy chain exhibited severe biventricular hypertrophy. At 8 months of age, only male mice displayed overt heart failure phenotype (76, 77). Also, transgenic mice with a missense mutation (R92Q) in the cardiac troponin T exhibited more severe fibrosis with marked hypertrophic marker gene induction in males, compared to females (78). Importantly, ovariectomized mutants showed worse phenotype of further impaired contractile function and myocardial energy metabolism, and estrogen supplementation restored these parameters (79), suggesting estrogen's protective effects against cardiac hypertrophy and heart failure in this model.

Studies using global deletion of ERα or ERβ exposed to chronic angiotensin II or pressure-overload have suggested the role for ERβ in the estrogen's protection against hypertrophy and failure, where calcineurin and cGMP-PKG signaling regulation might be significantly involved (80). In particular, the link of estrogen to the latter signaling pathway might be important and deserves further investigation. Myocardial cGMP-PKG signaling pathway is de-activated in human heart failure with preserved ejection fraction (HFpEF), while cGMP-PKG activation is regarded as a new therapeutic strategy to treating heart failure. Considering HFpEF is associated with female sex and increased age independent of obesity and diabetes, it is reasonable to speculate estrogen loss and subsequent cGMP deactivation might contribute to the pathophysiology of HFpEF. Importantly, Sasaki et al. reported that estrogen signal is required in order to activate cGMP-PKG using a PDE5 inhibitor in female cardiac myocytes to ameliorate heart failure in female mice (41, 81, 82). There are several other reported molecular mechanisms including a mammalian target of rapamycin signaling, regulation of phosphorylation of p38 mitogen-activated protein kinase pathway, and regulation of cardiomyocyte histone deacetylases (83–85).

### Vascular Injury and Atherosclerosis

Estrogen inhibits excessive responses to vascular injury in a mouse carotid artery injury model. Here, *in vivo* estrogen treatment inhibits the proliferation of VSMC and promotes re-endothelialization (53, 86–92). In ERβ KO mice, estrogen is still protective against vascular injury (86, 93), whereas, in ERα KO mice, estrogen treatment shows no protective effect on the vascular injury response (86, 90). This supports the concept that ERα is responsible for estrogen's protective effect on vasculature.

The role of the non-nuclear ER signaling pathway in estrogen-induced vascular protection was evaluated in a “gain of function” and “loss of function” study. EDC promoted carotid artery re-endothelialization in an ERα-dependent manner and diminished the development of neointimal hyperplasia following vascular injury in a manner equivalent to estrogen treatment (53). Of note, endometrial carcinoma cell growth *in vitro* and breast cancer xenograft growth *in vivo* were stimulated by estrogen, but not EDC (53). This suggests that selective activation of the non-nuclear signaling pathway did not promote cancer growth. Moreover, estrogen significantly decreases injury-induced neointimal hyperplasia while inhibiting VSMC proliferation in wild-type mice. Strikingly, estrogen had no significant effect in DPM mice (62). Taken together, non-nuclear signaling plays a major role in estrogen-induced protection against vascular injury, and compounds that activate non-nuclear signaling might provide a vascular benefit as non-nuclear selective ER modulators without increasing the risk of uterine or breast cancer.

Ligand-bound ERα mediates target gene transcription through the activation function 2 (AF2) domain, located on the C-terminal, and ligand binding domain of ERα. A recent report that used knock-in mice that lacked a functional AF2 domain showed that AF2 is needed to inhibit atherosclerosis (94). In contrast, the effect of estrogen on re-endothelialization after vascular injury was preserved in these mice (94), suggesting an essential role of the non-nuclear ER signaling on endothelial healing after vascular injury.

## Conclusion

Large-scale, randomized clinical studies from the early 2000s failed to demonstrate cardiovascular benefits associated with post-menopausal estrogen treatment, and rather showed an increased risk of breast cancer and thrombosis. However, recent clinical trials and meta-analyses have suggested that estrogen treatment has beneficial effects for preventing CVD and does not affect mortality if HT is initiated early after menopause (6, 11). In addition to the timing of HT initiation, various factors including treatment duration, dose, formulation, regimen (estrogen or estrogen plus progesterone), and route of administration may affect cardiovascular outcomes. Greater clarity of the molecular mechanisms by which estrogen regulates specific cardiovascular processes is needed to optimize next-generation HT.

Various ERs are expressed in most cardiovascular cell types, including cardiomyocytes, fibroblasts, vascular EC, and VSMC. Each ER is involved in protective effects of estrogen in multiple animal disease models, including ischemic heart disease, cardiac hypertrophy, heart failure, vascular injury, and atherosclerosis. Emerging evidence has indicated the potential importance of the non-nuclear ER signaling on diverse aspects of the cardiovascular systems, supporting the potential opportunity to design pathway-specific selective ER modulators capable of regulating non-nuclear and nuclear effects, assisting with the development of personalized therapies for preventing and treating CVD. Further research will provide more insight into therapeutic approaches that translate basic science findings into clinical practice innovations.

## Author Contributions

KU and ET wrote the manuscript. YA, PL, and NF critically revised the manuscript and contributed to designing the figures.

### Conflict of Interest

The authors declare that the research was conducted in the absence of any commercial or financial relationships that could be construed as a potential conflict of interest.
